# Intestinal TG3- and TG2-Specific Plasma Cell Responses in Dermatitis Herpetiformis Patients Undergoing a Gluten Challenge

**DOI:** 10.3390/nu12020467

**Published:** 2020-02-13

**Authors:** Hanna Sankari, Minna Hietikko, Kalle Kurppa, Katri Kaukinen, Eriika Mansikka, Heini Huhtala, Kaija Laurila, Timo Reunala, Kaisa Hervonen, Teea Salmi, Katri Lindfors

**Affiliations:** 1 Celiac Disease Research Center, Faculty of Medicine and Health Technology, Tampere University, 33520 Tampere, Finland; hanna.sankari@tuni.fi (H.S.); minna.hietikko@tuni.fi (M.H.); katri.kaukinen@tuni.fi (K.K.); kaija.laurila@tuni.fi (K.L.); kaisa.hervonen@tuni.fi (K.H.); teea.salmi@tuni.fi (T.S.); 2 Tampere Center for Child Health Research, Tampere University, Tampere, the Department of Paediatrics, Tampere University Hospital, 33520 Tampere, Finland; kalle.kurppa@tuni.fi; 3 The University Consortium of Seinäjoki and Seinäjoki Central Hospital, 60220 Seinäjoki, Finland; 4 Department of Internal Medicine, Tampere University Hospital, 33520 Tampere, Finland; 5 Department of Dermatology, Tampere University Hospital, 33520 Tampere, Finland; timo.reunala@tuni.fi; 6 Faculty of Social Sciences, Tampere University, 33520 Tampere, Finland; heini.huhtala@tuni.fi

**Keywords:** coeliac disease, dermatitis herpetiformis, transglutaminase, autoantibody, plasma cell

## Abstract

Dermatitis herpetiformis (DH), a cutaneous manifestation of coeliac disease, is characterized by transglutaminase (TG) 3-targeted dermal immunoglobulin A (IgA) deposits. The treatment for DH is the same as for coeliac disease, namely a life-long gluten-free diet. DH patients typically have gluten-dependent circulating autoantibodies targeting TG3 and TG2, and plasma cells secreting such autoantibodies have been detected in the small intestinal mucosa. This study investigates the gluten-responsiveness of intestinal TG3 and TG2 antibody-secreting plasma cells in 16 treated DH patients undergoing a gluten challenge. The frequency of both plasma cell populations increased significantly during the challenge, and their frequency correlated with the corresponding serum autoantibody levels at post-challenge. TG3-specific plasma cells were absent in all 18 untreated coeliac disease patients and seven non-coeliac control subjects on gluten-containing diets. These findings indicate that, in DH, both intestinal TG3- and TG2-antibody secreting plasma cells are gluten-dependent, and that TG3-antibody secreting plasma cells are DH-specific.

## 1. Introduction

Dermatitis herpetiformis (DH), a cutaneous manifestation of coeliac disease, is characterised by an itching and blistering rash predominantly on the elbows, knees, and buttocks that arises in response to the ingestion of gluten-containing cereals, i.e., wheat, rye, and barley. The key diagnostic feature for DH is the presence of granular immunoglobulin A (IgA) deposits in the papillary dermis, which are known to target an endogenous human protein, transglutaminase (TG) 3 [1]. Furthermore, in the majority of DH patients, IgA-class anti-TG3 autoantibodies are also found in the circulation [1,2]. The circulating TG3 autoantibodies are not entirely specific to DH, as approximately 30% of untreated coeliac patients have elevated levels of these autoantibodies in the absence of any skin symptoms [3,4]. In DH patients, the skin symptoms slowly resolve during a gluten-free diet, the well-accepted and effective treatment, but the disappearance of TG3-targeted IgA deposits from the skin takes many years despite the faster clearance of the serum TG3 autoantibodies [1,5,6,7,8,9].

As DH and coeliac disease are different manifestations of the same condition, they also share the major genetic susceptibility conferred by HLA-DQ2 or -DQ8 [10]. In addition, small bowel mucosal villous atrophy [11,12] or at least coeliac-type inflammatory changes are typically present also in untreated DH [13,14]. The major autoantigen in coeliac disease is TG2, a member of the TG family along with TG3, and untreated patients characteristically have TG2-targeting autoantibodies (e.g., TG2 and endomysial antibodies, EmA) in the circulation and within various tissues, including the small intestine; as deposits at the subepithelial basement membrane and around the blood vessels [15,16]. Gluten-dependent TG2 autoantibodies are commonly found also in DH both in the serum and in the small intestinal mucosa [17,18]. In untreated coeliac disease, TG2 antibody-secreting plasma cells are present in the small bowel at a high frequency, and their number decreases on a gluten-free diet [19,20,21]. We have recently established that TG3 antibody-secreting cells are present in the small bowel mucosa in DH [22], but no other studies have addressed intestinal TG3 or TG2 plasma cells in DH. Therefore, we investigated the frequency and gluten-responsiveness of both of these plasma cell populations in treated DH patients undergoing a gluten challenge, assessed their correlations with corresponding serum antibodies, and compared their presence in DH and coeliac disease.

## 2. Materials and Methods

### 2.1. Patients

The DH patient cohort included 11 males and 5 females, who were recruited on voluntary basis to a prospective gluten challenge study to investigate the possible development of gluten tolerance as described elsewhere [9]. At the time of recruitment, the patients were adhering to a gluten-free diet. In all patients, the DH diagnosis had been based on the typical clinical picture and the presence of granular IgA deposits in the papillary dermis as demonstrated with a direct immunofluorescence examination. At pre-challenge, the median age of the patients was 58 (range 37–72) years, and the patients had been on gluten-free diet for a median of 22 (range 5–40) years (Table 1). The inclusion criteria for the gluten challenge were clinical remission for at least three years, negativity for TG2 antibodies and EmA, and normal villous architecture in a duodenal biopsy. The gluten challenge comprised an initial three-day challenge with 200 g of commercially available wheat-based bread (equivalent to 10 slices) daily followed by a gluten-containing diet with a minimum of 10 g of wheat per day. Post-challenge examinations were performed upon the appearance of a DH rash or positive seroconversion (either TG2 antibodies or EmA), or after 12 months of the gluten challenge. Pre- and post-challenge investigations included skin and small intestinal biopsies as well as the determination of TG2 and TG3 antibodies and EmA in the patient’s serum.

The coeliac disease control group consisted of both untreated (*n* = 18) and treated (*n* = 15) patients having adhered to a gluten-free diet for one year (Table 1). The median age of the untreated patients was 50 (range 18–71) years; seven were male and 11 were female. The median villous height crypt depth ratio (Vh/CrD) was 0.3 (range 0.04–3.4). Fifteen (83%) patients were TG2 antibody-positive and 16 (89%) were EmA-positive; the median TG2 and EmA levels were 60 (range 3.1–101) U/mL and 1:1000 (range 0–1:4000), respectively. Seven (39%) of the untreated coeliac disease patients were TG3-antibody positive (median 7 AU/mL, range 0–189). As regards the 15 treated coeliac disease patients (median age 48 (range 19–72) years, 4 males), the median Vh/CrD was 2.6 (range 2.1–3.1). Three subjects were TG2 antibody-positive, and the median level in the group was 1.6 U/mL (range 0–26). Five subjects had EmA, and the median titre was 0 (range 0–1:200). Four (27%) of the patients were TG3-antibody positive (median 10 AU/mL, range 0–42).

In addition, seven patients investigated due to unspecific abdominal symptoms served as the non-coeliac disease control group (median age 47 (range 24–76) years, 6 females) (Table 1). Coeliac disease was excluded based on findings of normal small bowel mucosal histology and negative serum TG2 antibodies and EmA.

All subjects gave their informed consent for inclusion before they participated in the study. The study was conducted in accordance with the Declaration of Helsinki, and the protocol was approved by the Regional Ethics Committee of the Pirkanmaa Hospital District, Tampere, Finland (the ethic approval codes are R16039, R03041, R04097 and R07122).

### 2.2. Serology

Serum IgA-class EmA was determined as previously described, with a dilution of 1:≥5 being considered positive [23]. IgA-class TG2 and TG3 antibodies were detected using commercial enzyme-linked immunosorbent assay kits (ELISA) (Celikey^®^, Phadia, Freiburg, Germany, and anti-heTG IgA ELISA, Immunodiagnostik AG, Bensheim, Germany, respectively) as instructed by the manufacturers. The cut off for positivity used in the present study was as instructed in the manuals, namely ≥5 U/mL and >22 AU/mL for TG2 and TG3 antibodies, respectively.

### 2.3. Skin and Small Bowel Biopsies

Skin biopsies were taken from uninvolved elbow skin or perilesional skin when the rash was present. The biopsies were fixed in optimal cutting temperature compound (OCT, Tissue-Tec, Miles Inc. Elkhart, IN, USA), snap-frozen in liquid nitrogen, and stored at −70 °C until analysed. Cutaneous IgA deposits were detected in frozen sections by direct immunofluorescence staining using fluorescein isothiocyanate (FITC)-conjugated rabbit anti-IgA antibody (1:20, Dako A/S, Glostrup, Denmark). The deposits were graded as negative (0), weak (1), moderate (2) or strong (3). The colocalization of the dermal IgA with TG3 was demonstrated by double stainings with FITC-conjugated rabbit polyclonal TG3 antibody (1:100) (A030, Zedira, Darmstadt, Germany) and tetramethylrhodamine-isothiocyanate (TRITC)-conjugated goat anti-human IgA (1:50) (A18786, Life Technologies, Frederick, MD, USA). Small intestinal biopsies were taken from the duodenum upon upper intestinal endoscopy. For morphological studies, at least two biopsies were fixed in formalin, embedded in paraffin, and processed for haematoxylin and eosin staining. The Vh/CrD was determined as previously described [24], with a ratio of ≥2.0 being considered normal. For the detection of mucosal TG2-targeting IgA deposits, 1–2 biopsies were embedded in OCT and snap-frozen in liquid nitrogen. Sections were stained using mouse monoclonal anti-TG2 antibody (CUB7402; NeoMarkers, Fremont, CA, USA) and FITC-labelled rabbit anti-human IgA antibody (Dako A/S) as previously described [16]. The IgA deposits were graded as negative (0), weak (1), moderate (2) or strong (3).

### 2.4. Detection of TG3- and TG2-Specific Plasma Cells

For the determination of intestinal TG3 and TG2 antibody-secreting plasma cells, small intestinal biopsies were available from all 16 DH patients at pre-challenge and post-challenge, and from all 18 untreated and 15 treated coeliac disease patients, as well as the seven non-coeliac control subjects. Immunofluorescence staining of the plasma cells was performed using a technique previously described [19,21]. Briefly, 5 µm-thick frozen sections of small intestinal biopsies were air-dried for 20 min at room temperature (RT), washed with phosphate-buffered saline (PBS), and incubated with either in-house biotinylated recombinant human TG2 (2 µg/mL, T002, Zedira) or TG3 (5 µg/mL, T024, Zedira) in 1% bovine serum albumin-PBS (BSA-PBS) for 45 min at RT. Prior to staining, biotinylation of TG2 and TG3 was achieved with EZ-Link^®^ Sulfo-NHS-LC-Biotin (Thermo Scientific, Waltham, MA, USA). Thereafter, the sections were washed with PBS and incubated with TRITC-labelled streptavidin (1:1000, Invitrogen, Camarillo, CA, USA) in 10% BSA-PBS for 30 min at RT. To distinguish the plasma cells, sections were further incubated in the presence of mouse monoclonal anti-human CD138 antibody (1:25, Bio-Rad Antibodies, Oxford, UK) in 1% BSA-PBS for 45 min at RT, followed by Alexa Fluor 488-conjugated anti-mouse antibody (1:2000, Invitrogen) in 1% BSA-PBS for 30 min at RT.

The slides were viewed with an Olympus BX60F5 (Olympus Finland Oy, Espoo, Finland) microscope. The percentage of TG3- and TG2-specific plasma cells was determined by counting the number of TG3- and TG2-specific plasma cells and the overall number of plasma cells in the entire small bowel biopsy section by an independent evaluator guided by an expert consultant.

### 2.5. Statistics

The continuous variables are described with median values and ranges, and the categorized values are presented as numbers and percentages. Statistical analyses were performed using the Mann–Whitney *U* test, the Wilcoxon test, and Fisher’s exact test as appropriate. Spearman’s correlation coefficient was applied to assess correlations between different variables. A *p*-value < 0.05 was considered statistically significant. The analyses were performed using IBM SPSS Statistics for Windows (Version 23.0, IBM Corp., Armonk, NY, USA).

## 3. Results

### 3.1. General response to the gluten challenge in DH patients

After a median of four months (range 1–12 months) of gluten challenge, 12 out of the 16 DH patients developed the DH rash. In addition, the number of patients with dermal TG3-IgA deposits increased during the gluten challenge from 1 to 10. In parallel, the number of serum TG3 antibody-positive DH patients increased from 4 (median level 4, range 0–41 AU/mL) to 14 (median level 140, range 5–190 AU/mL) (Table 2). At pre-challenge, none of the patients had circulating TG2 antibodies or EmA, whereas at post-challenge, serum TG2 antibodies were present in 10 cases and EmA in 12 cases. The median TG2 antibody level was 101 (range 0–101) U/mL and that of the EmA titre 1:500 (range 0–1:4000). During the challenge, the median Vh/CrD decreased from 2.6 (2.1–4.5) to 0.8 (range 0.1–3.1). Intestinal TG2-IgA deposits were not detected in any of the DH patients at pre-challenge, but they were present in 10 cases at post-challenge.

### 3.2. TG3-specific plasma cell responses

**Figure 1:**
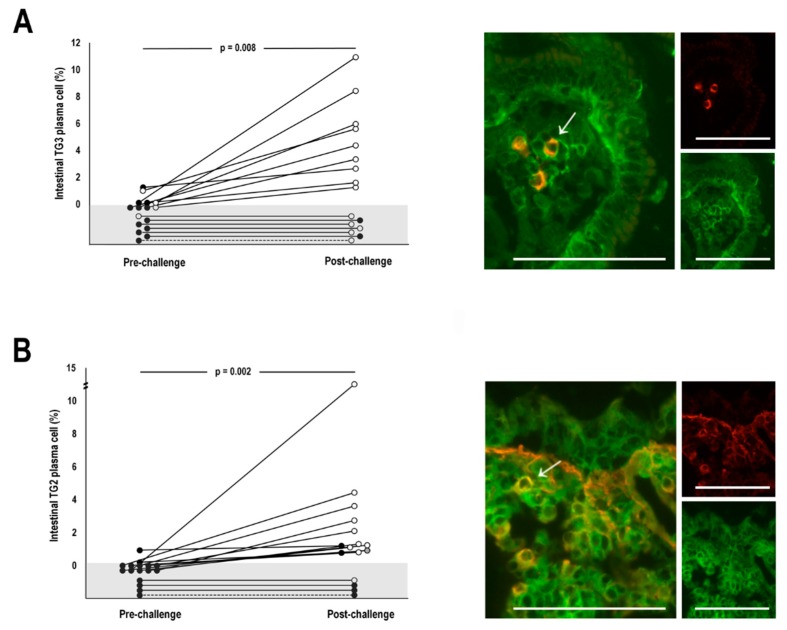
The percentage of transglutaminase (TG) 3- (**A**) and TG2-specific plasma cells (**B**) of all intestinal plasma cells in dermatitis herpetiformis patients at pre- and post-challenge. The grey area represents the absence of plasma cells. The black circles in A indicate negativity for serum TG3 antibodies and in B for both endomysial and TG2 antibodies, while the white circles indicate positivity. The grey circle in B indicates one individual with positive EmA but missing data for TG2 autoantibodies. Representative immunofluorescent pictures showing specific plasma cells (indicated with arrows) are shown on the right. The colour yellow indicates the colocalization of recombinant TG3 or TG2 (red) with plasma cell marker CD138 (green). Scale bar = 100 μm. A *p*-value < 0.05 was considered significant.

At pre-challenge, intestinal TG3 antibody-secreting plasma cells were detected in 2 out of 16 biopsy samples taken from DH patients (Figure 1A). The median percentage of such cells out of all lamina propria plasma cells in the whole pre-challenge group was 0 (range 0–1.2%). At post-challenge, these cells were found in 9 of the 16 DH patient mucosal specimens, and the median percentage in the group was 1.4 (range 0%–10.9%) (Figure 1A). To address the putative TG2-cross-reactivity of the TG3 antibodies secreted by the plasma cells, we preincubated the sections with unlabelled recombinant TG2 prior to biotinylated TG3 and found that the number of TG3 antibody-secreting cells was not reduced (data not shown). Regardless of the presence or absence of serum TG3 antibodies, neither the untreated coeliac disease patients nor the non-coeliac control subjects had intestinal TG3 antibody-secreting plasma cells, but they were present in one treated coeliac disease patient (1.9% of all lamina propria plasma cells).

Of the two DH patients with intestinal TG3 antibody-secreting plasma cells at pre-challenge, one was serum TG3 antibody-positive (30 AU/mL) (Figure 1A), whereas among the 14 patients without such plasma cells, three had circulating TG3 antibodies (23, 40, and 41 AU/mL). At post-challenge, regardless of the presence or absence of intestinal TG3 antibody-secreting plasma cells, all DH patients except two TG3 plasma cell-negative subjects had serum TG3 antibodies. The DH patients with TG3 antibody-secreting plasma cells at post-challenge had significantly higher circulating TG3 antibody levels than those without such cells (median 190 AU/mL (range 24–190) vs. 30 AU/mL (range 5–190), respectively, *p* = 0.023)). A correlation between the percentage of intestinal TG3 antibody-secreting plasma cells and serum TG3 antibody levels was detected (Rs = 0.541, *p* = 0.030) (Table 3).

All 9 DH patients with intestinal TG3 antibody-secreting plasma cells at post-challenge had skin symptoms compatible with DH, and seven of these had dermal TG3-IgA deposits. Of the seven patients without such plasma cells, three presented with the DH rash and dermal TG3-IgA deposits in the post-challenge examination. The intensity of the dermal TG3-IgA deposits and Vh/CrD did not differ statistically between the TG3 antibody-secreting plasma cell-positive and -negative DH patients at post-challenge, nor did they correlate with the percentage of intestinal TG3 antibody-secreting plasma cells (Table 3).

### 3.3. TG2-specific plasma cell responses

Intestinal TG2 antibody-secreting plasma cells were present in two DH patients at pre-challenge, and both were TG2-seronegative. The median percentage of TG2 antibody-secreting plasma cells in the whole group at pre-challenge was 0 (range 0%–0.9%) (Figure 1B). At post-challenge, 12 of the 16 DH patients had such plasma cells in the duodenal samples, and the median percentage of the cells had increased to 1.2 (range 0%–12.6%) (Figure 1B). In the coeliac disease control group, 17 of the 18 untreated patients had TG2 antibody-secreting plasma cells (median 4.2%, range 0%–20.0%), while such cells were found in 13 of the 15 treated coeliac patients (median 1.1%, range 0–4.6). Only one of the seven non-coeliac controls patients had intestinal TG2 antibody-secreting plasma cells (0.4% of all intestinal plasma cells).

Of the 12 DH patients with TG2 antibody-secreting plasma cells at post-challenge, 9 had TG2 antibodies in the serum, 11 had EmA in the serum, and 10 presented with intestinal TG2-IgA deposits. Of the four patients without TG2 antibody-secreting plasma cells, one had both TG2 and EmA, but none had TG2-IgA deposits in the small bowel mucosa. The patients with TG2 antibody-secreting plasma cells at post-challenge had significantly higher circulating TG2 antibody levels (median 101 U/mL (range 0–101) vs. 0 U/mL (range 0–9.5), *p* = 0.010)) and EmA (median 1:500 (range 0–1:4000) vs. 0 (range 0–1:200), *p* = 0.013)), as well as more intense intestinal TG2-IgA deposits (median 1.5 (range 0–3) vs. 0 (0 range), *p* = 0.013)) compared to the plasma cell-negative patients. Vh/CrD was comparable between the groups (median 0.8 (range 0.1–2.5) vs. median 0.8 (range 0.7–3.1), *p* = 0.862)). The percentage of intestinal TG2 antibody-secreting plasma cells correlated with the levels of serum TG2 antibodies (Rs = 0.759, *p* = 0.001) and EmA (Rs = 0.732, *p* = 0.001) as well as the intensity of the intestinal TG2 deposits (Rs = 0.691, *p* = 0.003), but not with Vh/CrD (Table 3).

## 4. Discussion

The current study confirms the presence of TG3 antibody-secreting plasma cells in the small intestinal lamina propria of DH patients [22]. Moreover, according to the current study—and also our previous study [22]—such cells can only be detected in single coeliac patients, pointing to the specificity of this cell population for DH. In addition, our data shows that in long-term treated DH patients in clinical remission, both the intestinal TG3 and TG2 antibody-secreting plasma cells are mostly absent, but in more than half of the patients the cells appear after a gluten challenge of up to one year. Therefore, the study is the first to address the dynamics of these cell populations during gluten reintroduction in DH.

According to our results, during a median of four months of gluten challenge, the percentage of TG3 and TG2 antibody-secreting plasma cells of all lamina propria plasma cells increased significantly. Although these plasma cell populations were not detected in all DH patients with a clear gluten-induced disease relapse, the findings strongly point to the gluten-dependency of these cells. In this regard, our results are in line with the previously reported gluten responsiveness of TG2-specific plasma cells in coeliac disease [19,21]. Earlier studies have reported TG2 antibody-secreting cells in all untreated coeliac disease patients with median values above 4% [19,20,21]. In addition, such intestinal TG2 antibody-secreting cells with median values around 2% have been described in a subset of early-phase coeliac disease patients with positive serum TG2-targeted antibodies but normal small bowel mucosal morphology [21]. Thus, the percentage of both TG2 and TG3 antibody-secreting plasma cells in DH patients after a gluten challenge in the current study is more comparable to early-phase coeliac disease rather than untreated coeliac disease. This fits well with the fact that small bowel mucosal damage in DH is often milder than in coeliac disease, and thus DH is often considered to represent incipient coeliac disease [25]. In the current study, the fairly short gluten exposure might have had an effect of the number of patients with the intestinal plasma cell subsets as well as the percentage of such cells, and a longer gluten intake might be required for all DH patients to react to a similar extent as in overt coeliac disease.

In the current study, the percentage of both intestinal TG3- and TG2-specific plasma cells at post-challenge correlated with the corresponding serum autoantibody levels in DH. This finding seems to contrast with the previously reported lack of correlation between the frequency of intestinal TG2 antibody cells with serum TG2 antibody levels in untreated coeliac disease [19,21,26]. However, the earlier reported data and our current results are not directly comparable; they are derived from different disease manifestations, and our new finding in DH patients represents the scenario of a rather unique reactivation of the gluten-induced immune response within a comparably short period. Among our cohort of DH patients, there were also TG3- and TG2-seropositive subjects without corresponding plasma cells in the intestine. This might reflect the fact that one biopsy might be unrepresentative for the duodenum and the whole small intestine as the mucosal lesion can be patchy or affect only certain parts of the bowel. Recently, the molecular composition of serum and intestinally produced TG2 antibodies have been shown to differ, suggesting that TG2 antibody-secreting plasma cells are at least not the sole source of corresponding serum autoantibodies [27]. Thus, our results that at subject level, the presence of intestinal TG3- and TG2-specific plasma cells did not always coincide with the serum antibodies could be explained by either of the two above mentioned scenarios.

Two distinct populations of TG3 antibodies have been suggested to exist: one targeting TG3 specifically and detected in only DH, and another recognizing both TG3 and TG2 that is present in both DH and coeliac disease patients [1]. Our results show that the number of intestinal TG3 antibody-secreting plasma cells was not reduced in DH patients by preincubation with recombinant TG2, suggesting that plasma cells secreting TG3-specific antibodies which are not cross-reactive with TG2 are present in the intestinal mucosa, and thus this fits well with the concept of DH patients having TG3-specific antibodies. On the other hand, we did not detect recombinant TG3-binding cells in the intestinal mucosa in the majority of coeliac patients—even those with serum TG3 antibodies—a finding congruent with earlier results demonstrating the lack of cross-reactivity of coeliac patient intestinal TG2 antibodies with TG3 [28]. Based on this, it appears that the intestinal TG3 antibody-secreting plasma cells are specific to DH. However, it must be noted that an immunofluorescence method might not be optimal for detecting cross-reactive antibody populations and thus more investigations regarding coeliac disease patient TG3 antibody-secreting cells as well as the origin of serum TG3 antibodies are needed. Despite their specificity for DH, according to our results some patients at post-challenge had clinical and/or immunological skin findings in the absence of intestinal TG3 antibody-secreting plasma cells. This implies that the TG3 autoantibodies produced specifically by intestinal plasma cells are neither invariably related to the presence of dermal IgA deposits, as previously implicated [29,30], nor to the appearance of cutaneous symptoms.

The strength of the current study is the well-defined cohort of long-term-treated DH patients followed-up prospectively for up to one year during a gluten-challenge. This design allowed us to investigate the dynamics of the plasma cells secreting TG3 and TG2 autoantibodies and to compare them to several other disease parameters in DH. It must be noted, however, that our findings might not be applicable to DH at the time of diagnosis, when the patients are likely to have ingested gluten for a considerably longer period. A further limitation is the relatively small number of serum TG3 antibody-positive coeliac disease patients, which hampers the drawing of definitive conclusions about this particular group. However, as only around 30% of coeliac patients are reported to have circulating TG3 autoantibodies [3], we consider our coeliac patients representative. Moreover, the untreated and one-year-treated coeliac disease patients are not directly comparable to the DH patients undergoing a gluten challenge.

## 5. Conclusions

The current study verifies for the first time that intestinal TG3 and TG2 antibody-secreting plasma cells are gluten-responsive in DH. Moreover, the percentage of both plasma cell subsets correlates with the levels of corresponding serum antibodies in DH patients after a gluten challenge, but the presence of plasma cells and serum antibodies does not always coincide at the individual patient level. Furthermore, the absence of intestinal TG3 antibody-secreting plasma cells in coeliac patients, even those with serum TG3 antibodies, points to the specificity of these cells for DH. Regardless of this disease specificity, their connection with other disease parameters, including skin symptoms and dermal IgA deposits, is weak, and therefore further studies on their significance in DH are needed.

## Figures and Tables

**Table 1:** Demographic data of patients participating the study.

	Dermatitis Herpetiformis *n* = 16	Coeliac Disease		Non-Coeliac Controls *n* = 7
		Untreated	Treated	
		*n* = 18	*n* = 15	
Females; *n* (%)	5 (31)	11 (61)	11 (73)	6 (86)
Age, years, median (range)	58 (37–72)	50 (18–71)	48 (19–72)	47 (24–76)
Duration of GFD at diagnosis, years, median (range)	22 (5–40)	0	1 (1–1)	0

GFD: gluten-free diet.

**Table 2:** Serological, skin, and small bowel mucosal biopsy findings in 16 gluten-challenged dermatitis herpetiformis (DH) patients at pre- and post-challenge.

	Pre-Challenge		Post-Challenge	
	Positive Cases, *n* (%)	Median (Range)	Positive Cases, *n* (%)	Median (Range)
Serum TG3 antibodies (AU/mL)	4 (25)	4 (0–41)	14 (88)	140 (5–190)
Dermal TG3-IgA	1 (16)	0 (0–1)	10 (63)	1 (0–3)
Serum TG2 antibodies (U/mL)	0 (0)	0 (0–0)	10 (63)	101 (0–101)
Serum EmA (titre)	0 (0)	0 (0–0)	12 (75)	1:500 (0–1:4000)
Intestinal TG2-IgA deposits	0 (0)	0 (0–0)	10 (63)	1 (0–3)
Vh/CrD		2.6 (2.1–4.5)		0.8 (0.1–3.1)

The cut-off value for positivity was ≥5 U/mL for serum TG2 antibodies, 1:≥5 for serum EmA, and >22 AU/mL for serum TG3 antibodies. The intensity of dermal TG3-IgA deposits and intestinal TG2-IgA deposits was graded from negative (0) to strong positive (3). TG2: transglutaminase 2; EmA: endomysial antibodies; Vh/CrD: villous height crypt depth ratio; TG3: transglutaminase 3; IgA: immunoglobulin A.

**Table 3:** Correlations between the percentages of intestinal transglutaminase (TG) 3- and 2-specific plasma cells and relevant serum, skin, and small bowel mucosal findings in dermatitis herpetiformis (DH) patients at post-challenge. R_S_, Spearman correlation coefficient. A *p*-value < 0.05 was considered significant.

	R_S_	*p*-Value
TG3-specific plasma cells		
Serum TG3 antibodies	0.541	0.030
Intensity of dermal TG3-IgA	0.175	0.517
Vh/CrD	−0.273	0.307
TG2-specific plasma cells		
Serum TG2 antibodies	0.759	0.001
Serum EmA	0.732	0.001
Intensity of intestinal TG2-IgA deposits	0.691	0.003
Vh/CrD	−0.195	0.469

TG3: transglutaminase 3; IgA: immunoglobulin A; Vh/CrD: villous height crypt depth ratio; TG2: transglutaminase 2; EmA: endomysial antibodies.
